# Sleep Dysfunction in COVID-19 Patients: Prevalence, Risk Factors, Mechanisms, and Management

**DOI:** 10.3390/jpm11111203

**Published:** 2021-11-14

**Authors:** Athanasia Pataka, Seraphim Kotoulas, Elpitha Sakka, Paraskevi Katsaounou, Sofia Pappa

**Affiliations:** 1 Respiratory Failure Unit, G Papanikolaou Hospital Medical School, Aristotle University of Thessaloniki, 57010 Thessaloniki, Greece; akiskotoulas@hotmail.com; 2 School of Pharmacy and Biomolecular Sciences, University of Brighton, Brighton BN2 4AT, UK; elpithasakka@hotmail.com; 3 Department of Respiratory Medicine, First ICU, National and Kapodistrian University of Athens, 15772 Athens, Greece; paraskevikatsaounou@gmail.com; 4 Department of Brain Sciences, Imperial College London, London W12 0NN, UK; s.pappa@imperial.ac.uk or; 5 West London NHS Trust, London UB2 4SD, UK

**Keywords:** COVID-19 patients, sleep dysfunction, mental health, insomnia, obstructive sleep apnea

## Abstract

During the COVID-19 pandemic, the need to establish the prevalence of sleep dysfunction and psychological distress, identify predisposing and protective factors, and explore effective management strategies remains an important priority. Evidence to date suggests that a considerable proportion of COVID-19 patients experience significant sleep disturbances (estimated to afflict up to 50–75%) as well as psychological distress such as depression, anxiety, and traumatic stress. Duration of hospitalization, pre-existing mental health concerns, lower absolute lymphocyte count, and increased neutrophil-to-lymphocyte ratio have been all associated with a greater risk of sleep dysfunction in infected and hospitalized patients. Furthermore, in this review, we discuss the link between sleep deprivation, susceptibility to viral infections, and psychosocial wellbeing in relevance to COVID-19 and summarize the existing evidence regarding the presence and role of sleep apnea in infected individuals. Finally, we highlight the importance of suitable interventions in order to prevent and manage sleep dysfunction and avoid long-term physical and psychological implications. Future research should aim to provide high-quality information including in high risk, underserved, or difficult to reach populations and on the long-term consequences and effectiveness of applied interventions.

## 1. Introduction

At the end of December 2019, a series of cases of a novel coronavirus (SARS-CoV-2), causing respiratory infections in humans were described in Wuhan, China. The virus spread rapidly worldwide causing the coronavirus disease 2019 (COVID-19). The World Health Organization (WHO) declared COVID-19 a ‘Public Health Emergency of International Concern’ on the 30 January 2020 and a ‘pandemic’ on the 11 March 2020 [1,2]. Social distancing, frequent hand hygiene, use of masks, and isolation with quarantine were all measures imposed to minimize the exposure to the virus in the absence of vaccines and successful treatment strategies [3].

Previous studies have shown that infectious disease outbreaks have been associated with disturbed sleep and psychological distress, i.e., traumatic stress, depression, and anxiety [4,5,6]. Contributing factors include physical illness, mental health history, environmental stressors, social isolation, and separation from family and friends, and other measures to control the outbreak [4,5,6]. As with previous infectious diseases, existing evidence suggests that the novel coronavirus disease has had a significant impact on public mental health [7] causing fear, mental health difficulties, and sleep disorders in different populations, including the general public, healthcare professionals (HCPs), and patients infected from SARS-CoV-2. According to a number of studies, sleep dysfunction and other neuro-psychiatric symptoms are particularly prevalent in patients with COVID-19 [8,9]. For example, post-traumatic stress disorder symptoms were reported in up to 96% of hospitalized stable patients, while levels of depression (29%) were also elevated among newly recovering patients compared with those in quarantine (9.8%). 

Moreover, some patients who have been infected with SARS-CoV-2may display prolonged new, recurring, or ongoing symptoms for more than four weeks after infection, sometimes even after an initial symptom recovery [10]. These health consequences have been grouped together under the term ‘post-COVID syndrome’ or ‘post-acute sequelae of COVID’ or ‘long COVID’. This condition can occur even in patients with mild or asymptomatic infections. It can be difficult to distinguish symptoms caused by post-COVID from those occurring for other reasons as patients experience social isolation due to mitigation measures and adverse psychological effects. In addition, the post-COVID-19 syndrome may overlap with multi-organ complications and may include the adverse effects of treatment or hospitalization, for example post-intensive care syndrome (PICS), deconditioning, severe weakness, and post-traumatic stress disorder (PTSD) [10]. Symptoms and clinical findings as fatigue, difficulty in concentration, persistent respiratory symptoms, loss of smell or taste, post-exertional malaise, depression, anxiety, and sleep disorders are commonly reported in the ‘post-COVID syndrome’ [10,11]. 

Therefore, the consequences of poor sleep in COVID-19 sufferers are multiple, varied, and far reaching. Here, we aim to summarize the prevalence of sleep dysfunction in the face of COVID-19, identify the risk factors for its development, and explore ways in which we can minimize its impact. In addition, we discuss the links between psychosocial stress, sleep deprivation, and susceptibility to viral infections in relevance to COVID-19 as well as the presence and role of obstructive sleep apnea (OSA). 

## 2. Sleep Dysfunction in COVID-19 Patients

### 2.1. Prevalence of Sleep Problems in COVID-19 Sufferers

Sleep is an important factor for human wellbeing in order to maintain daily functions [12], while lack of sleep may lead to an increase in accidents, mood changes, impaired psychological functioning and concentration, and decreased immune response rendering individuals more susceptible to contracting the virus [13]. Thus, several studies have focused on sleep problems during the COVID-19 pandemic mostly using self-reported data in different populations. In fact, the prevalence of COVID-19 related sleep disorders has been reported to be high, affecting approximately 30–35% of the general public and HCPs [14,15]. Patients suffering from active COVID-19 display an even higher prevalence of sleep problems [14,15]. In a recent systematic review and meta-analysis, the corrected pooled estimated prevalence of sleep problems was 57% among COVID-19 patients compared to 31% in HCPs and 18% in the general population [14]. 

In all groups studied, sleep problems were positively associated with psychological distress, such as depression and anxiety. Country of residence and lockdown period significantly influenced the development of sleep difficulties. However, the meta-regression analysis in this study indicated that factors such as age, gender, country, and marital status did not contribute to the prevalence of sleep problems. Furthermore, COVID-19 patients displayed the highest prevalence of sleep problems with HCPs having the second place [14], especially front-line clinical staff [15,16,17,18]. HCPs due to their job demands may suffer from a high level of sleep difficulties and stress as they need to be in frequent contact with infected patients and need to adapt to irregular and sometimes prolonged work schedules and shifts [18,19,20]. More specifically, the prevalence of insomnia has been found to be higher among non-medical HCPs (e.g., students, community workers, and volunteers) than among medical HCPs and among frontline HCPs compared to non-frontline HCPs [21,22]. 

Another recently conducted meta-analysis on this subject [15] also confirmed that the most affected group for sleep problems during the pandemic was patients with COVID-19 with a pooled prevalence rate of 74.8%, followed by HCPs (36.0%), and then by the general population (32.3%). The prevalence of sleep problems including all the populations from the available different studies (general population, HCPs, patients) was estimated at almost 36% [15]. The subgroup analysis by population showed that most of the studies evaluated the sleep problems of the general population, followed by those of HCPs, and the minority evaluated sleep problems of patients with SARS-COV-2. In the subgroup of COVID 19 patients, older age and male sex were associated with higher sleep problems prevalence [15]. However, a subgroup analysis in the study of Jahrami et al. showed that studies that included only female participants reported a higher prevalence of sleep problems [15], which is in accordance to prior evidence that female gender is related to higher risk for insomnia and mental health problems [23]. On the other hand, in a study assessing the mental health status and sleep quality of COVID-19 patients hospitalized during different pandemic stages in a single center in Wuhan [24], about half of the patients reportedly exhibited a mild level of depressive mood, especially female and elderly patients, indicating that gender might be an independent predictor for anxiety and depression status. Elderly patients and those with co-morbid chronic diseases were more likely to report sleep problems. Additionally, those patients who reported moderate or severe symptoms were more likely to suffer from sleep problems compared to those who only reported mild symptoms. The factor that was significantly associated with mental distress and disturbed sleep quality was patients’ subjective perception of disease severity rather than the objective clinical classification [24]. 

The definition of insomnia varied among the published studies during the pandemic; most referred to insomnia in its broader presentation, as insomnia disorder or insomnia symptoms, with limited information about its onset and maintenance (acute/transient, short term, chronic) [25]. Being an HCP or at risk of contact with COVID- 19 patients, being a woman, having co-morbidities, and living in rural areas were found to be the most important risk factors for insomnia [26]. Insomnia symptoms and fatigue were more frequently reported from COVID-19 patients during the recovery from acute infection, compared with shortness of breath [27] and depression [28]. In any case, effective management with the appropriate interventions of this disorder is crucial, as it may lead to severe consequences such as an increased risk of suicide and substance abuse [29,30]. 

The instrument used to estimate sleep problems in most of the studies (50%) was the Pittsburgh Sleep Quality Index (PSQI) [31]. The mean PSQI for all different populations examined in the meta-analysis of Jahrami et al. [15] was estimated to be 7.1 (95% CI, 6.3–8.0) with scores for the general population around 6.0 (95% CI, 5.3–6.8) and higher for HCPs with a score of 7.7. Sleep latency, sleep disturbances, and sleep duration estimated with the PSQI were affected the most, presenting with the highest scores [15]. Apart from PSQI, a range of other validated scales were used for the assessment of sleep disturbances such as Athens Insomnia Scale, Insomnia Severity Index (ISI) [32], alongside researcher-developed measures or subsections of an established questionnaire [15]. This range of different measures used in the studies to assess symptoms across different time frames has contributed to the large heterogeneity of results in the meta-analysis of the studies. For example, PSQI and Athens Insomnia Scale [33] evaluate sleep symptoms during the past month, whereas ISI evaluates sleep symptoms during the past 2 weeks. On the other hand, PSQI evaluates a very broad range of sleep disorders such as nightmares and snoring, which could explain the higher prevalence rates in the studies using this instrument compared with others such as ISI, which evaluate insomnia symptoms but do not evaluate other sleep disturbances. 

However, the pandemic is ongoing, and the results of different studies cannot be generalized. Nevertheless, it remains important to evaluate the impact of COVID-19 on sleep in different ethnicities, different age groups, and different strata of society, including those with limited access to health care services as well as the longer-term consequences [34]. Furthermore, most of the current studies did not investigate lifestyle factors such as smoking, substance use, physical activity, marital status, or employment; thus, their results should be interpreted taking these limitations into consideration. 

### 2.2. Sleep Impairment as a Neurological Symptom of SARS-CoV2

SARS-CoV2′s most frequent severe clinical finding is pneumonia, leading to acute respiratory distress syndrome (ARDS). However, mounting evidence has shown that it can affect the nervous system, causing neurological symptoms from the early phases of the disease [35]. Sleep impairment was found to be the most frequent neurological symptom, followed by headache, dysgeusia, hyposmia, and depression. Daytime sleepiness was more frequent in the first two days after admission, and sleep problems were observed more commonly in patients after 7 days of hospitalization. These patients also demonstrated higher white blood cells and lower C-reactive protein (CRP) levels. A gender-based difference was reported with women experiencing daytime sleepiness more frequently; this was attributed to the different immune responses to viral infections, which were more marked in women compared to men [36].

In a prospective study evaluating the neurologic disorders in hospitalized COVID-19 patients in New York, patients with new neurological complications during hospitalization were matched to COVID-19 patients without neurological complications and were followed up for 6 months [37]. Almost two-thirds of all patients reported fatigue, sleep disorders, depression, and anxiety. Moreover, poorer sleep quality was associated with acute respiratory failure requiring invasive mechanical ventilation. Over 90% of COVID-19 patients reported at least one abnormal outcome at 6 months follow up, with 62% experiencing worse than average anxiety, depression, fatigue, or sleep. Additionally, patients with neurological complications presented with significantly worse functional outcomes. 

In fact, there is evidence to support a link between coronavirus infections and various nervous system manifestations. Neuroinflammation has been also noted with COVID-19 but appears to play a role in other neuropsychiatric diseases, several of which are characterized by immune-inflammatory states, and their treatments may have anti-inflammatory properties and effects. The underlying mechanism of the psychiatric, neuropsychiatric, and sleep consequences of COVID-19 are multifactorial and may include the social isolation, the severity of viral infection, immunological reactions, different treatments applied as corticosteroids, the Intensive Care Unit (ICU) stay, and the social stigma [38].

### 2.3. Role of Sleep in the Immune Response to COVID-19

Sleep strongly affects the immune system, and there is evidence that sleep deprivation negatively impacts immune responses [39,40], possibly leading to immunosuppression [41]. However, the effects of sleep quality during hospitalization for COVID-19 remain unclear. Almost 15% of COVID-19 hospitalized patients reported somnolence [42]; however, it is not certain whether this was a consequence of factors such as pain, anxiety, constipation, physical discomfort from illness, or prolonged hospitalization or directly linked to COVID-19 [43,44]. A study [44] that objectively assessed with wrist actigraphy the consequence of severe symptoms of COVID-19 on sleep quality found that the patients with the most severe respiratory symptoms and those who required prolonged intensive care unit (ICU) stay presented lower Sleep Efficiency and Immobility Time and higher Fragmentation Index compared to those with mild respiratory symptoms not requiring ICU. In addition, a retrospective, single-center cohort study [45] conducted to investigate the effects of sleep, assessed by Campbell sleep questionnaire and PSQI, on recovery from lymphopenia and clinical outcomes of hospitalized COVID-19 patients found that patients with poor sleep recorded lower absolute lymphocyte count (ALC) and increased neutrophil-to-lymphocyte ratio (NLR). In COVID-19 patients with lymphopenia, poor sleep quality during hospitalization was associated with a slow lymphopenia recovery and an increased need for ICU admission. The reported slow recovery from lymphopenia and increased deterioration of NLR were associated with poor sleep quality during hospitalization for at least 2 weeks. 

These findings are suggestive of a potential close relationship between poor sleep quality during hospitalization and worse clinical outcomes in COVID-19 patients. This is further supported by evidence indicating that sleep deprivation has a substantial effect on immune cell number, function, and cytokine production. Lack of sleep may promote inflammatory factors release and impair human immunity [46], and both reduced and prolonged sleep time have been associated with a higher risk of respiratory infections [47,48]. Furthermore, insufficient sleep has been linked to illness severity with a study reporting that reduced sleep during the week prior to COVID-19 diagnosis was associated with more severe presentations and an inverse relationship between average daily sleep time before illness and severity of disease. In fact, the presence of co-morbidities and reduced sleep were the two most significant risk factors affecting the disease severity [49].

## 3. Sleep Impairment and Psychological Distress in COVID-19 Patients

High levels of psychological distress and poor mental health have been reported since the start of the COVID-19 pandemic in various populations [14,50,51]. Interestingly, a meta-analysis that summarized the prevalence of stress, anxiety, and depressive symptoms in the general population during COVID-19 reported prevalence rates of 29.6%, 31.9%, and 33.7% [52], respectively, which is overall comparable with the prevalence of sleep problems. These overlapping prevalence rates between psychological distress and sleep problems point toward a potential close relationship between sleep and neuropsychiatric co-morbidities. 

This bi-directional relationship is likely multifactorial. Thus, a high prevalence of sleep problems could be partly explained by psychological distress and sleep-related factors due to quarantine and lockdown as delayed bedtime and sleep onset as well as fear of COVID-19 and illness [53]. Fear of COVID-19 could be further exacerbated by the social media and exposure to news, including by the daily national and global COVID-19 death reporting [53]. In addition, COVID-19 positive status and older age were both factors that strongly correlated with both sleep disorders and psychological distress [15]. In a study evaluating the patients treated in a Fangcang Shelter Hospital in Wuhan 2 months after the beginning of the COVID-19 pandemic [54], factors that were related to the presence of anxiety and depressive symptoms included having a family member confirmed with COVID-19, symptom change after hospitalization, the number of current physical symptoms, and also poor sleep quality. In fact, almost 85% of the participants reported poor sleep, which was associated with more serious symptoms of anxiety and depression. In another cohort from Wuhan evaluating the consequences of COVID-19 after 6 months of symptoms onset, fatigue and muscle weakness were reported in 63%, anxiety or depression were reported among 23%, and sleep difficulties were reported in 26% of participants [55]. 

In addition, another systematic review and meta-analysis that aimed to assess the prevalence of depression, anxiety, and sleep disturbances of COVID-19 patients found that 45% of COVID-19 patients experienced depression, 47% experienced anxiety, and 34% experienced sleep disturbances [56]. Another study of patients suffering from schizophrenia showed poorer sleep quality and significantly higher stress, depression, and anxiety levels in the suspected COVID-19 group that was hospitalized in isolation [57]. Therefore, the above findings indicate that it is important to consider and address psychiatric co-morbidities when treating sleep problems in COVID-19 patients and vice versa [58]. Table 1 summarizes the most important findings of the studies assessing the sleep quality of COVID 19 patients. 

## 4. Obstructive Sleep Apnea (OSA) during COVID-19 Pandemic 

### 4.1. Association between OSA and COVID-19

Obesity, hypertension, diabetes, dyslipidemia, cardiovascular, and pulmonary disease have been found to be associated with severe outcomes from COVID-19 infection [59,60]. One of the most common co-morbidities among obese patients is obstructive sleep apnea (OSA), which is the most prevalent sleep-related breathing disorder. To date, several studies have shown a correlation between OSA and poor COVID-19 outcomes [61,62,63,64,65,66,67], whereas others found that patients with OSA had the same risk of contracting COVID-19 with that of non-OSA individuals [67]. Nevertheless, in a recent meta-analysis, OSA was found to be associated with worse COVID-19 outcomes and mortality, enhanced risk for more severe disease, need for mechanical ventilation, and ICU admission [68] (Table 2). 

This finding could be partly explained by the fact that OSA patients are often overweight or obese with multi-morbidity such as hypertension, diabetes, and cardiovascular disease, which all adversely affect COVID-19 related severity mortality outcomes [59,60]. Additionally, a dysregulation of the renin–angiotensin system (RAS) pathway has been observed [69] with increased angiotensin-converting enzyme (ACE) activity and higher levels of angiotensin II and aldosterone especially in OSA patients with pre-existing hypertension [70]. Furthermore, obesity influences RAS [71], which is involved in the pathogenesis of COVID-19 infection given that the virus uses ACE2 to enter into host cells; thus, RAS dysregulation in patients with OSA may promote the entry of the virus, resulting in more severe outcomes and higher mortality [68].

OSA also impairs sleep quality, causing sleep deprivation due to frequent awakenings from respiratory events. In turn, sleep deprivation has been shown to affect the immune system, increasing interleukins (IL-6, IL-17) and tumor necrosis factor-a (TNF-a), promoting inflammation [72,73] and possibly exacerbating the cytokine storm in COVID-19, which is related to ARDS [74]. Finally, the obstructive respiratory events result in intermittent hypoxia during sleep that could potentially aggravate the hypoxia from COVID-19 pneumonia, resulting in worse outcomes [66]. 

### 4.2. Management of OSA during COVID-19

Telemedicine may be used for the evaluation and follow up in order to minimize hospital visits and limit patients’ exposure to the virus. Patients diagnosed with OSA with suspected COVID-19 infection should be closely monitored for early detection and treatment to prevent severe outcomes. On the other hand, OSA diagnosis should be considered in COVID-19 patients with characteristic clinical findings. 

Various societies have issued guidelines regarding the safe function of sleep laboratories for the diagnosis and treatment of sleep disorders [75,76,77,78,79,80,81,82] and infection prevention [77]. Each sleep laboratory should adapt according to the local prevalence of COVID-19, increasing the levels of precautions when the community spread is high. Home sleep testing is preferred, and in-laboratory polysomnography should be performed on urgent and high-risk patients with disposable probes when available [78]. Symptoms and contact history for COVID-19 should be assessed in the case of an in-person appointment in the sleep laboratory with appropriate protective equipment such as surgical or FFP2 masks and gloves used. 

For the treatment of OSA patients during the COVID-19 pandemic, home positive airway pressure (PAP) should be applied using telemonitoring. General protective measures for patients and staff are important: disposable probes and non-leaky full facemask with a filter to the tubing is suggested. Additionally, during titration, humidifiers are not recommended [76]. All the equipment used should be sanitized according to the instructions of the manufacturer. The ventilator tube, all the sensors and equipment used should be carefully cleaned and disinfected with 75% ethanol or non-corrosive chlorine-containing disinfectant between each patient use [75,76,82].

## 5. Management of Sleep-Related Disorders in COVID-19 Patients

Evidently, there is a need for appropriate and tailored management strategies and interventions across different populations including the general public and high-risk groups such as HCPs and COVID-19 patients including improved sleep hygiene, identification of various risk factors at individual, interpersonal, institutional, and community levels, and early and accurate recognition of sleep dysfunction and psychological distress [30]. Special attention should be paid to COVID-19 patients, as there is a close association between sleep problems and psychological distress as well as sleep deprivation and COVID-19 severity and prognosis [44,45,46]. It has been suggested that improving the sleep quality of patients with mild or moderate lymphopenia in the early COVID-19 may promote the recovery of the immune function and possibly prevent the need for ICU admission [45]. In order to improve the sleep quality of hospitalized patients, it is important to adopt measures to reduce noise and lightening, to ensure privacy by separating patients from each other, and to provide psychological and emotional support as well as proper sedation and analgesia when needed. 

Medication strategies such as the administration of melatonin or sedating psychotropic medications have been also suggested in order to improve the sleep quality of COVID-19 sufferers [43,83,84]. Sleep deprivation is considered a risk factor but also a consequence of delirium: hence, chronotherapy, elimination of noise, and light exposure is recommended in order to decrease delirium in ICU [83,84]. On the other hand, diaries have shown limited benefit in the management of ICU delirium, depression, and post-traumatic stress [83].

The prophylactic administration of melatonin and melatonin receptor agonist, Ramelteon, improved the sleep of ICU patients, decreasing the prevalence of delirium and ICU length of stay, as it may have alleviated ARDS due to its anti-inflammatory and immune-enhancing effects [85]. Melatonin should be considered a first-line agent to treat sleep–wake rhythm disorders. Given its safety, melatonin may help to minimize the administration of medication as benzodiazepines or antipsychotics that may aggravate delirium or lead to central respiratory depression [85]. Melatonin has been also shown to improve acute lung injury in animal models acting as an antioxidant [43]. Ramelteon was found to have a protective effect against ventilator-induced lung injury, upregulating interleukin (IL-10) in rats [86,87]. High melatonin doses up to 10 mg have been administered to COVID-19 ICU patients for the prevention and treatment of delirium and sleep disturbances [85]. Further studies are needed in order to investigate the impact of higher doses in the reduction of the cytokine storm in patients with worse prognosis [43].

Additionally, a selective serotonergic agent fluvoxamine showed encouraging preliminary results in limiting disease progression in mild to moderate COVID-19 illness in a randomized trial [88], as it almost tripled the plasma levels of melatonin [89]. The use of other psychotropics may also be play a role in addressing sleep dysfunction and possibly the underlying neuroinflammation. In an observational study, chlorpromazine decreased the symptoms of COVID-19 in psychiatric patients versus caregivers [90]. Other studies have highlighted different results with anti- but also pro-inflammatory actions of antipsychotic and antidepressant medication (i.e., olanzapine, venlafaxine, citalopram, mirtazapine, amitriptyline, and others) that may affect inflammatory mechanisms such as cytokine levels [91]. However, more data from clinical trials are needed, as there are still questions about possible interactions among antiviral agents, dexamethasone, and psychiatric medications [91].

Insomnia should initially be treated according to the European Sleep Research Society and the American Academy of Sleep Medicine guidelines with cognitive behavioral therapy (CBT) [30,92]. Medications should be used in the treatment of chronic insomnia when CBT is unavailable or has failed, or in the co-existence of other psychiatric disorders as major depression [25,92]. There is evidence suggesting that CBT via telemedicine can be effective individually or in groups via telephone or videoconferencing [93]. Apart from CBT, other non-pharmacological approaches such as progressive muscular relaxation [30,94] and other alternative solutions as yoga may be useful. Special care should be given in pregnancy, in mental illness, in homeless individuals, etc. Additionally, circadian rhythm disorders, nightmares, and opioids use during sleep should be treated according to practice guidelines [30,95,96]. However, personalized treatment usually has the best results [94], and to this effect, an international collaboration is underway using standardized questionnaires to assess sleep disorders i.e., insomnia, sleep apnea, nightmares, fatigue, REM sleep disorder, and physiological factors in relationship to COVID-19 in multiple countries [97]. Table 3 summarizes the main aspects of management of sleep disorders in patients suffering from COVID-19 described in this review.

## 6. Conclusions

More than one year into the pandemic and despite the implicit limitations in the quality of available studies, the evidence points to the magnitude of effect of COVID-19 on the sleep and psychological wellbeing of the general population and high-risk groups [97,98]. Sleep problems appear to be rather common in COVID-19 patients and are related to higher levels of psychological distress such as traumatic stress, depression, and anxiety and worse coronavirus-related outcomes including severity and mortality. Findings can be utilized to advise targeted interventions, taking heed of identified risk factors and predictors of poor sleep and mental health. Effective programs for the treatment of sleep problems may lead to the reduction of psychological distress and vice versa, and improving the sleep quality of infected patients may improve their outcomes. Future research should aim to provide high-quality, inclusive information on the long-term implications and the effectiveness of applied interventions.

## Figures and Tables

**Table 1:** Main findings of the studies assessing the sleep quality of COVID-19 patients.

Author/Year	Sample	Type of Study	Aim	Results
Alimoradi, 2021 [14]	168 cross-sectional, four case-control, and five longitudinal design papers comprising 345,270 participants (general population, HCPs, and COVID-19 patients)	Systematic review and meta-analysis	Assess sleep problems during the COVID-19 pandemic and its relationship with psychological distress	The corrected pooled estimated prevalence of sleep problems was 57% among COVID-19 patients, 31% in HCPs, and 18% in the general population. Sleep problems were positively associated with psychological distress, such as depression and anxiety among HCPs, the general population, and COVID-19 patients.
Jahrami, 2021 [15]	44 papers, involving a total of 54,231 participants from 13 countries (general population, HCPs, and patients with COVID-19)	Systematic review and meta-analysis	Examine the impact of the pandemic on sleep problems prevalence	Global pooled prevalence rate of sleep problems among all populations was 35.7%. COVID-19 patients appeared to be the most affected group, with a pooled rate of 74.8%. HCPs and the general population had comparative rates of sleep problems, with rates of 36.0% and 32.3%, respectively. Older age and male sex were associated with higher sleep problems prevalence.
Jiang, 2021 [24]	202 patients who were hospitalized in different pandemic stages in a single center in Wuhan (109 on February and 93 on March 2020)	Cross-sectional study	Evaluate the mental distress and sleep quality of patients by questionnaires SAS, SDS, and PSQI	Elderly and those with co-morbid chronic disease were more likely to report sleep problems. Patients who reported moderate or severe symptoms were more likely to suffer from sleep problems compared to those who only reported mild. Patients’ subjective perception of disease severity rather than the objective clinical classification was more significant.
Liu, 2021 [27]	324 COVID 19 patients in Shenzhen during the recovery period	Retrospective cohort	Analysis of the epidemiological information, discharge summaries, and laboratory results of patients	Insomnia symptoms/headache (12%), anxiety (11.5%), and chest distress/breath shortness (8.2%) were reported.
Xu, 2021 [28]	121 confirmed COVID-19 patients, from Anhui Provincial Hospital and Anqing Hospital	On-site psychological investigation	Insomnia and depression symptoms were evaluated through the ISI and the CES-D in the recovered COVID-19 patients two weeks after discharge	2 weeks after discharge, COVID patients showed high prevalence (26.45%) of insomnia and a relatively low prevalence of depression (9.92%). Significant differences in insomnia and depression prevalence were found among COVID-19 patients with physical, mental impairment, and the need for psychological assistance. Age and health status were influencing factors for insomnia.
Liguori, 2020 [36]	103 patients hospitalized at the University Hospital of Rome “Tor Vergata”	Observational study	Evaluation of subjective neurological symptoms	Neurological symptoms occurred in more than 90% of patients (more frequently women). Sleep impairment (49.5%) was the most frequent symptom. Daytime sleepiness was more frequent in the first two days after admission (45.5%). Sleep impairment was more frequent in patients with more than 7 days of hospitalization (69.2%).
Fontera, 2021 [37]	4491 patients hospitalized with COVID-19 with 13.5% developing a new neurologic disorder	Prospective, multicenter, observational study	Evaluation of new neurologic disorders prevalence, in-hospital mortality, and discharge disposition between patients with COVID-19 with and without neurologic disorders	Patients with neurological complications had significantly worse functional outcomes Over 90% of COVID-19 patients had at least one abnormal outcome at 6 months: 62% had worse than average anxiety, depression, fatigue, or sleep, 56% had limited daily activities, 50% had abnormal cognition, and 47% were unable to return to work.
Vitale, 2020 [44]	4 cases, Lombardy Italy	Case series	Determining sleep quality with sleep diaries, PSQI, and wrist actigraphy	Patients with more severe respiratory symptoms, and those who required prolonged ICU stay presented lower Sleep Efficiency and Immobility Time and higher Fragmentation Index compared to those with mild respiratory symptoms.
Zhang, 2020 [45]	135 patients (60 in good-sleep group and 75 in poor-sleep group) Wuhan Union Hospital, China	Retrospective, single-center cohort	Investigate the effects of sleep quality on recovery from lymphopenia and clinical outcomes in hospitalized patients with laboratory-confirmed COVID-19 with RCSQ and PSQI	Patients in the poor-sleep group had lower absolute lymphocyte count and increased neutrophil-to-lymphocyte ratio. COVID-19 patients with lymphopenia presented poor sleep quality during hospitalization, and this was associated with a slow lymphopenia recovery and increased need for ICU admission. The reported slow recovery from lymphopenia and increased deterioration of NLR were associated with poor sleep quality during hospitalization for at least 2 weeks.
Huang, 2020 [49]	203 adults infected with COVID-19 and 228 uninfected adults in three Chinese provinces, with 80.7% of the infected and 82.5% of uninfected	Multicenter, retrospective cohort study	Discover how physical activity and lifestyle affect the epidemic as well as the disease severity and prognosis of COVID-19 patients by answering a doctor-administered telephone questionnaire on lifestyle	The risk of severe infection increased with decreased sleep status being 6.7 (95% CI = 2.138–21.181, *p* = 0.001) times higher for potentially appropriate sleep and 8.612 (95% CI = 1.913– 38.760, *p* = 0.005) for lack of sleep. Reduction in daily sleep time significantly increased severity of disease (*p* = 0.002).
Dai, 2020 [54]	307 patients admitted to Fangcang shelter hospitals	Cross-sectional study	Evaluate the prevalence and major influencing factors of anxiety and depressive symptoms among COVID-19 patients via an anonymous online questionnaire consisting of a set of items on demographic and clinical characteristics, the Self-Rating Anxiety Scale, Self-Rating Depression Scale, and PSQI	Poor sleep quality and having ≥two current physical symptoms were independent risk factors for anxiety. 84.6% of the participants reported poor sleep, which was associated with more serious symptoms of anxiety and depression.
Huang, 2021 [55]	1733 of 2469 discharged patients with COVID-19 from Jin Yin-tan Hospital (Wuhan, China)	Ambidirectional cohort study	Describe the long-term health consequences of patients who have been discharged and investigate the associated risk factors with a series of questionnaires for evaluation of symptoms and health-related quality of life, physical examination, 6-min walking test, and blood tests	Fatigue or muscle weakness (63%) and sleep difficulties (26%) were the most common symptoms. Anxiety or depression was reported among 23% of patients.
Deng, 2021 [56]	31 studies (n = 5153)	Systematic review and random-effects meta-analysis	Assess the prevalence of depression, anxiety, and sleep disturbances in COVID-19 patients	The pooled prevalence of sleep disturbances was 34% (95% CI: 19–50%, I^2^ = 98%), of depression 45% (95% CI: 37–54%, I^2^ = 96%), and of anxiety 47% (95% CI: 37–57%, I^2^ = 97%).
Liu, 2020 [57]	21 hospitalized patients with schizophrenia with suspected COVID-19 in the isolation ward of a mental health hospital in Wuhan and 30 hospitalized patients with schizophrenia in the general ward of another mental health hospital in Yichang, China (clean group)	Retrospective study	Explore the clinical characteristics of hospitalized patients with schizophrenia with suspected COVID-19. PSQI scores used for sleep quality	Compared with patients in the clean group, patients in the COVID-19 suspected group had significantly higher PSQI scores (8.0 ±3.8 vs. 4.7 ±3.6, *p* = 0.005).

HCPs = health care professionals, SAS = Self-Rating Anxiety Scale, SDS = Self-Rating Depression Scale, PSQI = Pittsburgh Sleep Quality Index, ISI = Insomnia Severity Index, CES-D = Center for Epidemiology Scale for Depression, OR = odds ratio, CI = confidence interval, ICU = intensive care unit, NLR = neutrophil-to-lymphocyte ratio, RCSQ = Richards–Campbell sleep questionnaire.

**Table 2:** Main findings of the studies assessing OSA in COVID-19 patients.

Author/Year	Sample	Type of Study	Aim	Results
Cade, 2020 [61]	4668 patients 443 participants (9.5%) with sleep apnea	Observational study	Analyze electronic health record data from a large New England health care system to assess whether sleep apnea is an unrecognized risk factor for COVID-19-related death, hospitalization, ventilator use, and ICU admission among COVID-19 patients	Participants with sleep apnea had an increased all-cause mortality rate (11.7%) compared with controls (6.9%) (OR = 1.79; 95% CI 1.31–2.45, *p* < 0.001). Weaker associations were observed between sleep apnea and the composite outcome of ICU admission, mechanical ventilation, or death, or for hospitalization. Participants using CPAP in the prior year displayed a non-significant trend for attenuated composite outcome results (OR = 0.97, 95% CI 0.73–1.30) compared with participants without CPAP (OR = 1.23, 95% CI, 0.82–1.84).
Kar, 2021 [62]	213 patients	Prospective observational study	Estimate the prevalence of OSA in COVID-19 patients using various screening questionnaires (STOP-BANG, Berlin Questionnaire, NoSAS, and Epworth Scale) and assess OSA effect on outcome of disease	Possible OSA could be an independent risk factor for poor outcome in COVID-19 patients.
Maas, 2021 [63]	9405 participants with COVID-19 infections, 3185 (34%) were hospitalized, and 1779 (19%) with respiratory failure	Observational study	Evaluate the risk for COVID-19 diagnosis, hospitalization, and respiratory failure associated with OSA, and assess potential association between OSA, COVID-19 hospitalization, and progression to respiratory failure from the data of an electronic medical record system of 10 hospitals in the Chicago metropolitan area.	OSA was more prevalent among patients requiring hospitalization (15.3% versus 3.4%, *p* < 0.0001; OR 5.20, 95% CI (4.43, 6.12)) and those who progressed to respiratory failure (19.4% versus 4.5%, *p* < 0.0001; OR 5.16, 95% CI (4.41, 6.03)). After adjustment for BMI, hypertension, and diabetes, OSA was associated with increased risk for hospitalization (OR 1.65; 95% CI (1.36, 2.02)) and respiratory failure (OR 1.98; 95% CI (1.65, 2.37)).
Iannella, 2021 [64]	96 patients Group 1 (72 patients): hospitalized patients undergoing conventional oxygen therapy Group 2 (24 patients): patients requiring enhanced respiratory support	Observational retrospective study	Evaluate the association between the severity of COVID-19 respiratory illness and the risk of developing OSA using STOP-BANG	41.6% of COVID-19 patients in group 2 presented STOP-BANG scores between 5 and 8 (high risk of OSA) compared with 20.8% of group 1. Linear regression correlated a high value of STOP-BANG with lower values of PaO2/FiO2 ratio. Patients with PaO2/FiO2 ratio <300 presented more frequently a STOP-BANG value >3 (*p* = 0.03). Patients at risk of OSA could develop more severe respiratory disease due to COVID-19.
Feuth, 2020 [65]	28 patients	Retrospective cohort study	Explore baseline characteristics to identify risk for severe disease and critical care admission in Turku University Hospital	OSA was present in 29% of patients admitted for COVID-19, suggesting that it is an important risk factor for severe COVID-19.
Memtsoudis, 2020 [66]	124 severely and critically ill patients with respiratory failure (ICU n = 60, non-ICU n = 64)	Observational study	Explore if OSA is a potential contributor to high morbidity amongst severely and critically ill COVID-19 patients with respiratory failure	A potential contributor to the high morbidity amongst obese patients might be the high prevalence of undiagnosed OSA (11.4% in ICU patients).
Strausz, 2021 [67]	260,405 Finnish individuals from FinnGen Data Freeze 6 with 445 patients with COVID-19	Observational study	Study if OSA is an independent risk factor for COVID-19 infection or for severe COVID-19	8.5% had OSA. From 91 severe COVID-19 cases, 20.9% suffered from OSA. OSA patients have the same risk of contracting COVID-19 with non-OSA individuals. OSA patients had 2.93 times higher risk of being hospitalized due to COVID-19 than non-OSA individuals.
Hariyanto, 2021 [68]	21 studies with 54,276 COVID-19 patients	Systematic review and meta-analysis	Analyze the relationship between OSA and poor outcomes of COVID-19	OSA was associated with poor outcome (OR 1.72 (95% CI 1.55–1.91), *p* < 0.00001), severe COVID-19 (OR 1.70 (95% CI 1.18–2.45), *p* = 0.005), ICU admissions (OR 1.76 (95% CI 1.51–2.05), *p* < 0.00001), need for mechanical ventilation (OR 1.67 (95% CI 1.48–1.88), *p* < 0.00001), and mortality (OR 1.74 (95% CI 1.39–2.19), *p* < 0.00001).

OSA = obstructive sleep apnea, OR = odds ratio, CI = confidence interval, BMI = Body Mass Index

**Table 3:** Summary of the management of sleep disorders of COVID-19 patients [30,43,44,75,76,77,78,79,80,81,82,84,85,86,87,88,89,90,92,93,94,95,96,97].

Prevention	Early and accurate recognition of sleep dysfunction and psychological distress
	Personalized treatment
Improve sleep hygiene	Adopt measures to reduce noise and lightening
	Chronotherapy
	Ensure privacy by separating patients from each other during hospitalization
	Provide psychological and emotional support
	Provide proper sedation and analgesia when needed
Medication strategies	Melatonin and melatonin receptor agonists
	Melatonin should be considered as a first-line agent to treat sleep–wake rhythm disorders
Insomnia	CBT, even via telemedicine
	2. Progressive muscular relaxation and other alternative solutions as yoga
	3. Medications should be used in the treatment of chronic insomnia when CBT is unavailable or has failed or in the co-existence of other psychiatric disease
Obstructive sleep apnea (OSA)	Each sleep laboratory should adapt according to the local prevalence of COVID-19
	Home sleep testing is preferred in uncomplicated patients
	During the COVID-19 pandemic, home PAP could be applied using telemonitoring in uncomplicated patients
	Telemedicine may be used for the evaluation and follow up

CBT = Cognitive behavioral therapy PAP = positive airway pressure.

## Data Availability

Not applicable.
